# Biosynthesis and Characteristics of Aromatic Polyhydroxyalkanoates

**DOI:** 10.3390/polym10111267

**Published:** 2018-11-14

**Authors:** Manami Ishii-Hyakutake, Shoji Mizuno, Takeharu Tsuge

**Affiliations:** 1 Bioplastic Research Team, RIKEN Center for Sustainable Resource Science, 2-1 Hirosawa, Wako, Saitama 351-0198, Japan; 2 Department of Materials Science and Engineering, Tokyo Institute of Technology, 4259 Nagatsuta, Midori-ku, Yokohama 226-8501, Japan; mizuno.s.ai@m.titech.ac.jp

**Keywords:** polyhydroxyalkanoate (PHA), aromatic polymer, biodegradable polyester, mechanical property, thermal property, glass transition temperature (*T*_g_)

## Abstract

Polyhydroxyalkanoates (PHAs) are polyesters synthesized by bacteria as a carbon and energy storage material. PHAs are characterized by thermoplasticity, biodegradability, and biocompatibility, and thus have attracted considerable attention for use in medical, agricultural, and marine applications. The properties of PHAs depend on the monomer composition and many types of PHA monomers have been reported. This review focuses on biosynthesized PHAs bearing aromatic groups as side chains. Aromatic PHAs show characteristics different from those of aliphatic PHAs. This review summarizes the types of aromatic PHAs and their characteristics, including their thermal and mechanical properties and degradation behavior. Furthermore, the effect of the introduction of an aromatic monomer on the glass transition temperature (*T*_g_) of PHAs is discussed. The introduction of aromatic monomers into PHA chains is a promising method for improving the properties of PHAs, as the characteristics of aromatic PHAs differ from those of aliphatic PHAs.

## 1. Introduction

Polyhydroxyalkanoates (PHAs) are microbial polyesters produced by numerous bacteria, including *Ralstonia eutropha* and *Pseudomonas putida*. PHAs are accumulated as an intercellular energy and carbon storage material under nutrient-limited conditions in the presence of excess carbon sources [1,2]. PHAs exhibit valuable characteristics, such as biodegradability, biocompatibility, and thermoplasticity, and therefore can be used for medical, agricultural, and marine applications [3,4].

PHAs are partially crystalline polymers. Therefore, their thermal properties are generally expressed in terms of the glass transition temperature (*T*_g_) of the amorphous phase and the melting temperature (*T*_m_) of the crystalline phase [5]. The most common type of PHA, poly(3-hydroxybutyrate) [P(3HB)], has a *T*_m_ of 177 °C and *T*_g_ of 4 °C [6]. The thermal properties of P(3HB) are similar to those of polypropylene; however, P(3HB) is a highly crystalline material with poor elasticity. Additionally, P(3HB) shows secondary crystallization at room temperature, which means that its physical properties change depending on the aging time [7,8]. These characteristics limit the practical uses of P(3HB).

The physical and mechanical properties of PHAs are dependent on the types of monomers and monomeric composition. To date, about 150 different hydroxyalkanoic acids have been reported as constituent monomers of biosynthesized PHAs [9,10]. Based on monomer structure, PHAs are divided into three groups; short-chain-length PHAs (scl-PHAs) comprising 3–5 carbon atoms like P(3HB), medium-chain-length PHAs (mcl-PHAs) comprising 6–14 carbon atoms, and long-chain-length PHAs (lcl-PHAs) comprising more than 14 carbon atoms [11]. These PHAs show different thermal and physical properties. Generally, mcl-PHAs show lower *T*_m_ and *T*_g_ and more flexibility compared with scl-PHAs. Alteration of the monomer types and/or the composition of PHAs could lead to desirable polymer properties. For example, P(3HB-5 mol % 3-hydroxyhexanoate) [P(3HB-5 mol % 3HHx)] has a *T*_m_ of 138–147 °C and *T*_g_ of 0 °C [12], whereas medium-chain-length PHAs (mcl-PHAs) with C6–C12 are elastomers with a *T*_g_ between −53 and −28 °C and *T*_m_ between 45 and 69 °C [13].

In 1990, Fritzsche et al. [14] reported the production of an aromatic PHA, P(3-hydroxy-5-phenylvalerate) [P(3H5PhV)], from 5-phenylvaleric acid (5PhV) by *Pseudomonas oleovorans.* That was the first report of a biosynthesized PHA bearing an aromatic group as a side chain [14]. Recently, wide varieties of aromatic monomers have been introduced into biosynthesized PHA chains. These aromatic PHAs are attractive, not only in terms of novelty, but also for their possible functionality conferred by the benzene ring.

The purpose of this review is to summarize the types of biosynthesized PHAs bearing aromatic groups as side chains and their properties. Specifically, the effect of aromatic monomers on the *T*_g_ of PHAs is demonstrably different from that of other mcl-monomers, and is thus described in detail. Incorporation of aromatic monomers into PHAs is one possible method of improving the properties of PHAs by conferring superior physical properties, surpassing those of aliphatic PHAs. Additionally, the aromatic side groups do not compromise the important characteristics of PHAs, including their biodegradability, biocompatibility, and thermoplasticity. Thus, it is proposed that the introduction of aromatic monomers into PHA chains is a promising method for producing PHAs with improved material properties.

## 2. Biosynthesized PHAs Bearing Aromatic Groups as Side Chains

**Figure 1:**
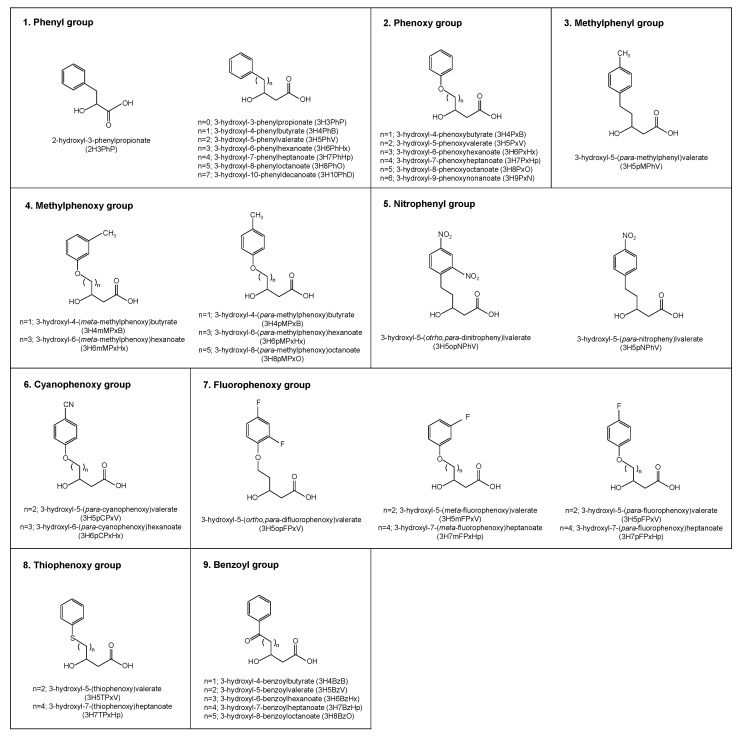
Structures of aromatic monomer units introduced into biosynthetic polyhydroxyalkanoates (PHAs). A monomer containing a nitrophenoxy group was also reported, but the detailed structure, including the carbon number, was not presented [24].

The production of a PHA containing an aromatic monomer was reported for the first time by Fritzsche and coworkers [14]. Thereafter, various aromatic monomers have been introduced into PHA chains through biosynthesis. Figure 1 shows the aromatic monomers incorporated into biosynthesized PHAs, and Table 1 summarizes the cultivation conditions used for the production of PHAs containing these monomers (the details are presented below).

Incorporation of most of the monomers shown in Figure 1 into PHA chains was achieved using *Pseudomonas* strains. Although there are some reports of the production of aromatic PHAs using recombinant *Escherichia coli* and *R. eutropha* [15,16,17,18], these cases also required PHA synthase from *Pseudomonas*. To the best of our knowledge, only PHA synthase from *Pseudomonas* can polymerize aromatic monomers.

Generally, to generate aromatic PHAs, defined compounds containing aromatic groups are added to the medium as a precursor (i.e., 5PhV or 5-phenyl-2,4-pentadienoic acid is used as a precursor to produce PHAs containing 3H5PhV [14,15,19,20]). However, there are a few reports on the production of aromatic PHAs without any supplementation with these defined aromatic compounds [16,18]. Therefore, aromatic PHAs could be divided into two groups, products synthesized from corresponding aromatic compounds through chemo-bioprocesses and products synthesized through complete biosynthesis from biomass. It was also reported that *Pseudomonas* strains fed with aromatic compounds produced PHAs composed of aliphatic monomers only [14,21,22,23]. 

### 2.1. Aromatic PHAs Produced from Corresponding Aromatic Compounds

As mentioned above, most biosynthesized aromatic PHAs have been produced from defined compounds with chemical structures similar to those of the incorporated aromatic monomers. Using various aromatic compounds as a precursor, many types of aromatic monomers containing phenyl, phenoxy, methylphenyl, methylphenoxy, nitrophenyl, nitrophenoxy, cyanophenoxy, fluorophenoxy, thiophenoxy, or benzoyl groups have been incorporated into PHA chains. The details are described below.

#### 2.1.1. PHAs Containing Phenyl Group

As monomer units of biosynthesized PHA, a number of monomer structures containing phenyl groups have been reported, including 3-hydroxy-3-phenylpropionate (3H3PhP), 3-hydroxy-4-phenylbutyrate (3H4PhB), 3-hydroxy-5-phenylvalerate (3H5PhV), 3-hydroxy-6-phenylhexanoate (3H6PhHx), 3-hydroxy-7-phenylheptanoate (3H7PhHp), 3-hydroxy-8-phenyloctanoate (3H8PhO), and 3-hydroxy-10-phenyldecanoate (3H10PhD). To the best of our knowledge, the incorporation of 3-hydroxy-9-phenylnonanoate into the PHA chain has not been reported. On the other hand, the incorporation of 2-hydroxy-3-phenylpropionate (2H3PhP) was reported [16,18]. The polymerization of these monomers resulted in PHA homopolymers or copolymers.

##### Homopolymer

*P. oleovorans* produced the P(3H5PhV) homopolymer from 5PhV [14]. This is the first example of a biosynthesized PHA containing an aromatic substituent. The same polymer was obtained when *P. putida* BM01 [19] or *P. putida* KT2440 [15] was used as the PHA production host. It was reported that the P(3H5PhV) homopolymer was also produced from 5-phenyl-2,4-pentadienoic acid by *P. oleovorans* [20] and from 7PhV or 9PhN by *P. putida* U [25]. In the latter case, the 3H5PhV monomer would be generated through the *β*-oxidation cycle. *P. putida* U also produced the P(3H6PhHx) homopolymer from 6-phenylhexanoic acid (6PhHx) [25,39,40].

##### Copolymer Composed of Monomers Bearing Phenyl Groups with Different Carbon Numbers

*P. putida* U produced random copolymers P(3H5PhV-3H7PhHp) and P(3H6PhHx-3H8PhO) from 7-phenylheptanoic acid (7PhHp) and 8-phenyloctanoic acid (8PhO), respectively [25,26]. It was reported that *P. putida* U produced the P(3H5PhV) homopolymer from 7PhHp [25]. These two different results might be derived from the method used for polymer analysis. This strain also produced a terpolymer containing 3H6PhHx, 3H8PhO, and 3H10PhD monomer units from 10-phenyldecanoic acid (10PhD) [25]. Monomers with different carbon numbers may be generated from the precursors through the *β*-oxidation cycle of the host strains, where the cycle is involved in fatty acid degradation. Similar results are documented in other reports [19,40]. Production of a terpolymer and tetrapolymer has also been reported [40].

##### Copolymer Containing Aliphatic and Aromatic Monomers

Copolymers composed of aliphatic and aromatic monomers have been reported. *P*. *oleovorans* cultured with 5PhV and *n*-alkanoic acid (octanoic acid (OA) or nonanoic acid (NA)) produced PHAs containing both 3H5PhV and aliphatic monomers [27]. The produced polymer was not a random copolymer, but comprised a mixture of two PHAs, and thus showed two different *T*_g_s (see Section 3). Similar results have been documented in other reports [27,41]. Transmission electron microscopy (TEM) observation demonstrated that these two polymers were formed in the same granule [41]. Therefore, there might be some distinguishing factor in the metabolism of the two substrates which results in the production of two separate polymers with different physical properties [41]. On the other hand, *P. citronellolis* and *P. putida* GPo1 produced a PHA random copolymer composed of aromatic and aliphatic monomers from a mixture of aromatic and aliphatic compounds [28,29].

It was also reported that PHA copolymers consisting of aromatic and aliphatic monomers were produced from phenylalkanoic acid as the sole carbon source. When 6PhHx, 7PhHp, or 8PhO was fed as the precursor, *Pseudomonas* strains accumulated PHAs containing predominantly aromatic monomers (3H4PhB, 3H5PhV, 3H6PhHx, 3H7PhHp, and 3H8PhO) with traces of aliphatic monomers (3-hydroxyoctanoate (3HO) and 3-hydroxydecanoate (3HD)) [22]. Similar results were obtained in other studies [15,32,42]. However, PHAs produced from styrene or phenylacetic acid by these strains contained only aliphatic monomers (3HHx, 3HO, and 3HD) without any aromatic monomers [22].

In the cases presented above, aromatic monomers were copolymerized with medium-chain-length (C6–C12) aliphatic monomers only, because typical PHA synthases from *Pseudomonas* (i.e., *P. putida*) are unable to polymerize the 3HB monomer. The copolymerization of 3HB and an aromatic monomer (3H3PhP) was achieved [15] using PHA synthase from *Pseudomonas* sp. 61-3 (PhaC1_Ps_) that could polymerize not only medium-chain-length 3HA (C6–C12), but also 3HB (C4) [43]. Recombinant *R. eutropha* PHB^-^4 expressing PhaC1_Ps_ generated P(3HB-3H3PhP) and P(3HB-3H4PhB) from a racemic mixture of 3-hydroxy-3-phenylpropionic acid plus fructose, and 4-phenylbutyric acid (4PhB) plus fructose, respectively [15,17]. These copolymers showed higher *T*_g_s than the P(3HB) homopolymer, unlike other 3HB-based copolymers such as P(3HB-3HHx) that showed lower *T*_g_s than the P(3HB) homopolymer [12] (see Section 3).

Recombinant *E. coli* expressing mutated PhaC1_Ps_ synthesized PHA copolymer containing 2H3PhP (phenyllactate) from amino acids such as phenylalanine and sugars [16]. Surprisingly, as described in Section 2.2, the same PHA copolymer was produced from sugars as the sole carbon source without amino acid supplementation [16,18].

#### 2.1.2. PHAs Containing Phenoxy Group

Incorporation of aromatic monomers containing the phenoxy group (C4–C9) was also reported [30]. *P. putida* BM01 produced a PHA composed of 3-hydroxy-5-phenoxyvalerate (3H5PxV) as the major component and 3-hydroxy-7-phenoxyheptanoate (3H7PxHp) as the minor component from 11-phenoxyundecanoic acid. Interestingly, 3-hydroxy-9-phenoxynonanoate (3H9PxN) was incorporated only when OA was added to the medium in conjunction with the aromatic compound. The production of PHAs containing the phenoxy group was also observed when *P. oleovorans* and *P. putida* were fed with *ω*-phenoxyalkanoic acids [31,44,45]. Similar to PHAs containing phenyl groups, these PHAs are highly or totally amorphous (see Section 3).

#### 2.1.3. PHAs Containing Methylphenyl Group

To produce a crystalline aromatic PHA, the synthesis of PHAs containing the methylphenyl group was attempted based on the results of previous studies [46,47,48]. These studies reported that the polymer formed by the cationic polymerization of *para*-methyl-α-methylstyrene showed a high degree of crystallinity, whereas the polymer obtained from *α*-methylstyrene did not crystallize. As described in Section 3, the obtained methylphenyl-containing PHA was crystalline.

As a precursor, methylphenylalkanoic acid could be introduced into PHA chains as an aromatic monomer. Poly(3-hydroxy-5-(*para*-methylphenyl)valerate) (P(3H5pMPhV)) was produced from 5-(*para*-methylphenyl)valeric acid by *P. oleovorans* [21]. Copolymerization of 3H5pMPhV and aliphatic monomers, including 3-hydroxyheptanoate (3HHp) and 3-hydroxynonanoate (3HN), was reported [32] using *P. putida* fed with 9-(*para*-methylphenyl)nonanoic acid as the sole carbon source.

#### 2.1.4. PHAs Containing Methylphenoxy Group

PHAs containing methylphenoxy groups were produced by feeding *P. putida* with methylphenoxyalkanoic acid as a carbon source. *P. putida* produced a random copolymer containing 3-hydroxy-4-(*para*-methylphenoxy)butyrate (3H4pMPxB), 3-hydroxy-6-(*para*-methylphenoxy)hexanoate (3H6pMPxHx), and 3-hydroxy-8-(*para*-methylphenoxy)octanoate (3H8pMPxO) from 8-(*para*-methylphenoxy)octanoic acid [33]. Similar monomers, 3-hydroxy-4-(*meta*-methylphenoxy)butyrate (3H4mMPxB) and 3-hydroxy-6-(*meta*-methylphenoxy)hexanoate (3H6mMPxHx), were also polymerized when 8-(*meta*-methylphenoxy)octanoic acid was supplied. However, no aromatic monomer was detected in the polymers isolated from the cells grown with 8-(*ortho*-methylphenoxy)octanoic acid. In other studies, the polymer produced from 8-(*ortho*-methylphenoxy)octanoic acid by *P. putida* KCTC2407 also did not contain any corresponding aromatic units [45]. These results indicate that the biosynthesis of PHAs bearing methylphenoxy substituents is highly dependent on the position of the methyl substitutent. Additionally, the position of methyl substitution also affects the crystallinity of the PHA (see Section 3).

#### 2.1.5. PHAs Containing Nitrophenyl or Nitrophenoxy Group

The production of aromatic PHAs bearing mononitrated or dinitrated groups has been reported [34]. In that study, to obtain a new bacterial PHA, a modified version of 5PhV containing the nitro group was fed to *P. oleovorans*. The bacteria eventually produced yellow PHAs with 1.2–6.9% repeating units containing *para*-nitro and/or *ortho*,*para*-nitrophenyl rings from 5-(*ortho*,*para*-dinitrophenyl)valeric acid and NA. However, the content of the dinitrated monomer unit decreased depending on the cultivation time. The bacteria initially synthesized a polymer containing *ortho*- and *para*-substituted aromatic groups, and then generated the polymer containing aromatic groups with only *para*-substitution. In fact, the final polymer showed two different *T*_g_s (see Section 3), indicating that the sample comprised a mixture of two PHAs [34]. Kim et al. reported the production of a PHA containing the nitrophenoxy monomer from 6-(*para*-nitrophenoxy)hexanoic acid and OA (the detailed monomer structure, including the carbon number, was not reported) [24]. Both studies showed that the extent of the incorporation of these monomers into the PHA was significantly lower than that of other aromatic groups, and -feeding of NA or OA along with the aromatic compounds was inevitably required.

#### 2.1.6. PHAs Containing Cyanophenoxy Group

An aromatic monomer containing hyperpolarizable cyanophenoxy side groups was also reported. *P. putida* KT2440 grown on OA and 6-(*para*-cyanophenoxy)hexanoic acid produced a PHA containing a monomer bearing the cyanophenoxy group, 3-hydroxy-6-(*para*-cyanophenoxy)hexanoate (3H6pCPxHx) [35]. Thermal analysis revealed that the produced polymer was not simply a random copolymer, but was a heterogeneous polymer consisting of various chains and/or chain segments with different compositions of 3H6pCPxHx units. The generated polymer showed weak second harmonic generation (SHG) signals [35]. The incorporation of cyanophenoxy groups, including 3-hydroxy-5-(*para*-cyanophenoxy)valerate (3H5pCPxV), was also reported by Kim et al. [24]. Similar to PHAs containing nitrophenyl or nitrophenoxy groups, co-feeding of OA was required to incorporate this monomer into the PHA chains.

#### 2.1.7. PHAs Containing Fluorophenoxy Group

Generally, polymers with fluorine atoms exhibit unique properties, including thermal and surface properties [49,50]. Therefore, attempts have been made to produce PHAs bearing the fluorophenoxy group. It was reported that *P. putida* produced PHAs with fluorinated phenoxy side groups from fluorophenoxyalkanoic acids [36]. P(3-hydroxy-5-(*meta*-fluorophenoxy)valerate-10.5 mol % 3-hydroxy-7-(*meta*-fluorophenoxy)heptanoate) [P(3H5mFPxV-10.5 mol% 3H7mFPxHp)], and P(3-hydroxy-5-(*para*-fluorophenoxy)valerate-8.7 mol % 3-hydroxy-7-(*para*-fluorophenoxy)heptanoate) [P(3H5pFPxV-8.7 mol % 3H7pFPxHp)] were produced from 11-(*meta*-fluorophenoxy)undecanoic acid and 11-(*para*-fluorophenoxy)undecanoic acid, respectively. When 11-(*ortho*,*para*-difluorophenoxy)undecanoic acid was used as the carbon source, the P(3-hydroxy-5-(*ortho*,*para*-difluorophenoxy)valerate) [P(3H5opFPxV)] homopolymer was produced. These PHAs showed water-shedding properties and higher thermostability than other aromatic PHAs (see Section 3).

#### 2.1.8. PHAs Containing Thiophenoxy Group

PHAs containing the thiophenoxy group were produced by *P. putida* 27N01 cultivated with thiophenoxyalkanoic acid [37]. This strain produced white cream-colored PHA copolymer containing 3-hydroxy-5-thiophenoxyvalerate (3H5TPxV) as the primary monomer unit and 3-hydroxy-7-thiophenoxyheptanoate (3H7TPxHp) as the minor unit from 11-thiophenoxyundecanoic acid. The produced polymer was amorphous, as described in Section 3.

#### 2.1.9. PHAs Containing Benzoyl Group

*P. cichorii* YN2 produced a PHA containing 3-hydroxybenzolyalkanoate units from benzoylalkanoic acids [38]. In that study, 3-hydroxy-4-benzoylbutyrate (3H4BzB), 3-hydroxy-5-benzoylvalerate (3H5BzV), 3-hydroxy-6-benzoylhexanoate (3H6BzHx), 3-hydroxy-7-benzoylheptanoate (3H7BzHp), and 3-hydroxy-8-benzoyloctanoate (3H8BzO) were incorporated into the PHA chains with 3HA units. The produced polymers had a hard texture at room temperature.

### 2.2. Aromatic PHAs Produced through Complete Biosynthesis

As described above, 2H3PhP-containing PHA was synthesized from phenylalanine and sugars [16]. Because phenylalanine could be produced from biomass in bacterial cells, it can be viewed that this PHA could be biosynthesized from biomass through complete biosynthesis. Similarly, it could be concluded that 3H3PhP-containing PHA could also be produced through complete biosynthesis because it was produced from sugars and cinnamic acid [15], which could be synthesized in bacterial cells. In this system, cinnamic acid is thought to be enantioselectively hydrated after ligation with CoA, and polymerized into the PHA chain. In fact, it was reported that 2H3PhP-containing PHA was produced from sugars as the sole carbon source [16,18]. These are rare reports showing the production of aromatic PHAs without any supplementation of corresponding aromatic compounds. Additionally, it is also suggested that 3H4PhB- or 2-hydroxy-4-phenylbutyrate (2H4PhB)-containing PHAs may be produced by complete biosynthesis, because homophenylalanine, which has a carbon skeleton identical to that of 3H4PhB, was reported to be biosynthesized from phenylalanine [51].

## 3. Physical and Chemical Properties of Aromatic PHAs

Aromatic PHAs show various characteristics depending on the types of aromatic monomers incorporated. Particularly, the thermal properties of aromatic PHAs have been extensively studied, demonstrating structure-specific behavior. In this section, the physical and chemical properties of aromatic PHAs are summarized.

### 3.1. Appearance

The physical appearance of aromatic PHAs varies depending on the types of incorporated monomers. PHAs composed of phenyl monomers only (P(3H5PhV)) [42,52] or phenoxy monomers only are sticky and soft [30]. In the case of P(3HA-3H5PhV), the polymer became soft with an increase in the 3H5PhV content [52]. P(3HA-3-hydroxy-*ω*-phenylalkanoate) [P(3HA-3HPhA)] became glue-like as the acyl chain length of the supplied *ω*-phenylalkanoic acid was increased [42].

PHAs bearing methylphenoxy groups are white, brittle materials [33,45]. Similarly, PHAs containing the 3H4BzB unit are relatively hard [38]. On the other hand, PHAs bearing thiophenoxy groups are cream-colored and elastomeric [37]. PHAs containing the difluorophenoxy monomer are also generally cream-colored [36].

In the case of PHAs bearing the nitrophenyl group, even with the introduction of a small amount of nitrophenyl units (1.2–6.9%), the physical properties became very different from that of mcl-PHA [34]. The polymer appeared yellow and had an elastic texture, whereas mcl-PHA produced from 3HN is whitish and sticky.

### 3.2. Mechanical Properties

The mechanical properties of P(3-hydroxydodecanoate-3H5PhV) [P(3HDD-3H5PhV)] with various 3H5PhV contents is summarized in Table 2 [52]. Introduction of the 3H5PhV unit into P(3HDD) resulted in a decrease in the yield strength, maximum tension strength, and elongation at break. Interestingly, P(3HDD-18.70 mol % 3H5PhV) showed a higher elongation at break than P(3HDD). On the other hand, the Young’s modulus became higher than that of P(3HDD), except for P(3HDD-31.97 mol % 3H5PhV). These results indicate a non-linear relationship between the 3H5PhV content and the mechanical properties.

### 3.3. Surface Properties

The surface properties of P(3H5opFPxV) with two fluorine atoms were evaluated [36]. The surface contact angle of this polymer was 104°, whereas that of the PHAs with a phenoxy or alkyl group (C3 and C5) in the side chain was approximately 50°. In general, a surface contact angle of over 100° is sufficient to allow utilization of the polymer as a non-wetting material. Therefore, this difluorinated PHA possessed water-shedding properties.

### 3.4. Degradability

The degradability of aromatic PHAs has also been studied. Degradability is one important property for the use of PHAs as biodegradable materials. For medical applications such as drug delivery systems, the stability of PHAs at physiological pH and the safety of the material released through hydrolysis should be evaluated by evaluating the chemical degradation. For agricultural and marine applications, degradation by microorganisms should also be studied.

#### 3.4.1. Chemical Degradation

The chemical degradation of the P(3H6PhHx) homopolymer was analyzed, as reported in the literature [40]. This polymer is quite stable around pH 7.0. Therefore, it could be used as a drug vehicle [54] to achieve slow release of the active product. Additionally, its hydrolytic products, which can be *β*-oxidized in vivo to phenylbutyric acid, phenylacetic acid, or *trans*-cinnamic acid, may exert important pharmaceutical effects, thereby improving or broadening the clinical effects of the encapsulated drug. Olivera et al. synthesized polymeric microspheres of P(3H6PhHx) at atmospheric pressure by a solvent evaporation method, and demonstrated its potential use as a drug vehicle [40].

The antibacterial activity of (*R*)-3-hydroxy-*ω*-phenylalkanoates (C5–C8), a hydrolytic product of PHAs bearing a phenyl group, is established [55]. The relevant study showed that all (*R*)-3-hydroxy-*ω*-phenylalkanoates inhibited the growth of *Listeria* species, attributed only (or mainly) to the (*R*)-enantiomer.

#### 3.4.2. Biological Degradation

Although aromatic PHAs are unusual in nature, some reports claim that they can be degraded by microorganisms such as *Pseudomonas* strains [27,35,56]. However, aromatic PHAs are degraded more slowly than PHAs having no aromatic repeating units. Interestingly, the rate of degradation of P(3H5PhV) became much greater when aliphatic PHA produced from 3HN was also present in the same cell, whereas the rate of degradation of aliphatic PHAs was not significantly affected by the presence of P(3H5PhV) [57]. Aliphatic PHA and P(3H5PhV) were degraded by the same depolymerase, and the enzyme worked more efficiently in the presence of aliphatic PHAs [57].

### 3.5. Solubility and Solvent Fractionation

Generally, bacterial PHA copolymers exhibit a broad comonomer compositional distribution, which may arise from changes in the bacterial metabolism during PHA biosynthesis. In addition, as shown in Section 2, biosynthesized aromatic PHAs are sometimes produced as a mixture of two different PHAs, but not simply as a copolymer. In some studies, these aromatic polymers were separated by solvent fractionation [17,27].

As reported [17], P(3HB-3H3PhP) and P(3HB-3H4PhB) were fractionated into several fractions with a narrow comonomer compositional distribution by using a chloroform/*n*-hexane mixture. The content of aromatic units in the fractionated samples increased as the concentration of *n*-hexane increased. In contrast, the molecular weights of the fractionated samples decreased as the concentration of *n*-hexane increased. This means that the samples containing less of the aromatic monomer with a high PHA molecular weight are difficult to dissolve in the chloroform/*n*-hexane mixture compared with the samples containing more of the aromatic monomer with a low PHA molecular weight. On the other hand, as reported [45], the sample with a larger content of aromatic monomers was isolated as an insoluble fraction. This means that the sample with a higher aromatic monomer content showed lower solubility in *n*-hexane than the sample containing less of the aromatic monomer. Similarly, an aromatic PHA was obtained as a precipitated fraction from solvent fractionation of a blend of aliphatic and aromatic PHAs by using a methanol/chloroform mixture [30]. The resulting precipitated sample had the same monomer composition as that of the aromatic PHA before blending, and no aliphatic monomers. From these results, it was suggested that solvent fractionation depended mainly the PHA molecular weight, which varied with the ratio of aromatic monomers.

### 3.6. Thermal Properties

PHAs are partially crystalline polymers. Therefore, their thermal properties are normally expressed in terms of the *T*_g_ of the amorphous phase and the *T*_m_ of the crystalline phase [5]. The thermal properties of the aromatic PHAs determined in various studies are summarized in Table 3. The properties differ significantly from that of mcl-PHAs, which are elastomers with *T*_g_s between −53 and −28 °C and a *T*_m_ between 45 and 69 °C [13], where the values vary according to the types of aromatic monomers.

#### 3.6.1. Thermal Properties of PHAs Containing Phenyl Groups

##### Homopolymer

P(3H5PhV), the first biosynthesized PHA bearing an aromatic group, has a *T*_g_ of 13 °C [14]. This is higher than that of PHAs with *n*-alkyl pendant groups (mcl-PHA), which have *T*_g_s in the range of −53 to −28 °C [13]. The increases of *T*_g_ resulted from the introduction of aromatic monomers have been also reported in other aromatic monomers as described below. This behavior is different from mcl-PHAs. On the other hand, the *T*_m_ of P(3H5PhV) ranged from 54 to 69 °C, which is similar to those of the *n*-alkyl-substituted PHAs. As for the value of *T*_m_, P(3H5PhV) is most similar to mcl-PHAs. However, the endothermic peak for the melting transition in the differential scanning calorimetry (DSC) thermogram was very small. This observation indicates that this polymer has a very low degree of crystallinity and is thus highly amorphous [20,27,52]. This is unusual because the homopolymer has a highly ordered isotactic structure, which generally provides an ordered packing structure in the solid state [21]. P(3H5PhV) showed another unusual feature whereby recrystallization occurred rapidly when the polymer was cooled rapidly from the melt, unlike the case of *n*-alkyl-substituted PHAs [14].

##### Copolymers Composed of Monomers Bearing Phenyl Groups with Different Carbon Numbers

The P(3H4PhB-95 mol % 3H6PhHx) copolymer has a *T*_g_ of 10 °C, which is higher than that of mcl-PHA, but lower than that of P(3H5PhV) [19]. Similar results were observed in another study [15]. The lower-temperature *T*_g_ may reflect decreased intermolecular interaction between the backbone chains, thereby resulting in increased backbone chain mobility, caused by lengthening of the side-chains by one methylene unit relative to that of 3H5PhV [58]. This sample did not exhibit any endothermic melting peak and was totally amorphous [15,19]. This suggests that the phenyl group as the side chain impedes the formation of a crystalline domain, probably due to steric hindrance [19].

Similarly, other PHAs composed of only monomers bearing phenyl groups produced from phenylalkanoic acids showed the same characteristics, being totally amorphous and with a higher *T*_g_ than that of mcl-PHA [26,40]. In the literature [26], the *T*_g_ values followed the order: P(3H5PhV-38 mol % 3H6PhHx-50 mol % 3H7PhHp) > P(3H5PhV-77 mol% 3H7PhHp) > P(3H6PhHx-73 mol % 3H8PhO). This is attributed to the increasing flexibility of the PHA chains due to the introduction of structural irregularities and alkyl side chains longer than three methylene units.

##### Copolymers Containing Aliphatic and Aromatic Monomers

PHAs consisting of 3HA and 45 mol % 3H5PhV as aliphatic and aromatic monomers, exhibited only one *T*_g_ at −20 °C [28]. On the other hand, another PHA consisting of 3HA and 40.6 mol % 3H5PhV showed two *T*_g_s at −31 and 5 °C [27]. Because these values are close to the *T*_g_ of the polymer produced from NA alone and P(3H5PhV), this sample is deduced to be a mixture of two PHAs. As described in Section 2, some biosynthesized aromatic PHAs are produced as a mixture of two polymers. In that case, the sample has two *T*_g_s.

The effect of incorporation of the phenyl monomer into P(3HA) on the thermal properties has been studied, and the effects of the types of aromatic monomers were investigated [42]. *P. putida* produced P(3HA-98 mol % 3H5PhV) and P(3HA-15 mol % 3H4PhB-83 mol % 3H6PhHx) from 5PhV and 6PhHx, respectively, where the PHAs were partially crystalline with *T*_m_s of 51.5 and 52.1 °C, respectively [42]. However, the PHAs produced from 7PhHp (P(3HA-85 mol % 3H5PhV-13 mol % 3H7PhHp)), 8PhO (P(3HA-7 mol% 3H4PhB-61 mol% 3H6PhHx-30 mol% 3H8PhO)), and 10PhD (P(3HA-6 mol % 3H4PhB-57 mol % 3H6PhHx-26 mol % 3H8PhO-9 mol % 3H10PhD)) showed no *T*_m_, indicating that these polymers were totally amorphous. The *T*_g_ of these polymers varied between 13.2 °C for P(3HA-98 mol % 3H5PhV) accumulated from 5PhV to −14.3 °C for P(3HA-7 mol % 3H4PhB-61 mol % 3H6PhHx-30 mol % 3H8PhO) accumulated from 8PhO. The decomposition temperature decreased as the length of the acyl side chain of the aromatic substrate was increased (283 °C for P(3HA-98 mol % 3H5PhV) produced from 5PhV to 254 °C for P(3HA-6 mol % 3H4PhB-57 mol % 3H6PhHx-26 mol % 3H8PhO-9 mol % 3H10PhD) from 10PhD) [42].

The effects of the aromatic monomer ratio were also investigated [52]. With an increase in the 3H5PhV ratio in the P(3HDD-3H5PhV) copolymer from 0 to 100 mol %, the *T*_g_ increased from −49.3 to 5.90 °C and the *T*_m_ decreased from 82.4 to 50.40 °C. Therefore, the thermal properties of the P(3HA-3HPhA) copolyester can be easily modified by changing the monomer content, which leads thermal properties intermediate between those of the two homopolymers.

The effect of the content of 3H5PhV in P(3H5PhV-3HA-3-hydroxy-*ω*-alkenoates(3HE)) was analyzed by Hartmann and coworkers [29]. Similar to the observations of Shen et al. [52], the *T*_g_ increased linearly from −38.7 to −6.0 °C as the 3H5PhV content increased from 0 mol % (P(3HA-10 mol % 3HE)) to 59 mol % (P(3HA-10 mol % 3HE-59 mol % 3H5PhV)). The estimated *T*_g_ of P(3H5PhV) calculated from these values is about 14.1 °C, and is in good agreement with the literature value (13 °C) [14].

The thermal properties of PHAs composed of 3HB and phenylalkanoic monomers were also determined [15,17]. The *T*_m_ of P(3HB-3H3PhP) with 4.1 mol % and 8.9 mol % 3H3PhP was similar to that of P(3HB) with Δ*H*_m_ = 7.1 − 27.8 J·g^−1^, which is an index of the degree of crystallinity, suggesting that these samples still contained the P(3HB) crystal phase. However, these values are lower than those of other copolymers, such as P(3HB-3HA) with 6 mol % 3HA (C6–C8) with a *T*_m_ of 126 °C and Δ*H*_m_ of 31 J·g^−1^ [59], and P(3HB-5 mol % 3HHx) with a *T*_m_ of 138 °C and Δ*H*_m_ of 45 J·g^−1^ [12]. This suggests that the phenyl side group of the 3H3PhP monomer strongly inhibited the crystallization of P(3HB). In fact, P(3HB-3H3PhP) with 15–21 mol % 3H3PhP did not show any melting behavior [17], indicating the lack of a crystal phase. The *T*_g_s of P(3HB-3H3PhP) and P(3HB-3H4PhB) were in the range of 9–20 °C and 7–10 °C, respectively, where these values are substantially higher than that of P(3HB) (5 °C) [17]. This is unusual behavior compared with that of aliphatic comonomers such as 3HV, 3HHx, and 3-hydroxy-4-methylvalerate (3H4MV). Although 3H4MV has a bulky iso-propyl structure and does not co-crystallize with 3HB [60], the *T*_g_ decreased as the 3H4MV content increased. The rigidity of the aromatic rings in the 3H3PhP and 3H4PhB monomers may account for these differences. The *T*_m_ of P(3HB-8 mol % 3H4PhB) is similar to that of low-density polyethylene, one of conventional plastics. The *T*_g_ of this PHAs (10–15 °C) is higher than that of low-density polyethylene (−30 °C), but it should be increased more to avoid secondary crystallization.

#### 3.6.2. Thermal Properties of PHAs Containing Phenoxy Groups

PHAs containing phenoxy groups are highly or totally amorphous with *T*_g_s higher than 10 °C [30,31,38,44]. The PHA composed of 3H5PxV and 3H7PxHp had a *T*_m_ of 70 °C with small Δ*H*_m_ (2.9 J·g^−1^), indicating that it is highly amorphous [30]. In another study, PHAs composed of 3-hydroxy-*ω*-phenoxyalkanoic acid (C4–C8) did not show any crystalline melting endotherm, indicating that these polymers were totally amorphous [31].

#### 3.6.3. Thermal Properties of PHAs Containing Methylphenyl Groups

P(3H5pMPhV) containing the methylphenyl group as a side chain is a crystalline polymer with a *T*_g_ of 18 °C and *T*_m_ of 95 °C with a defined endothermic peak [21], whereas P(3H5PhV) with no methyl substituent on the phenyl group is highly amorphous. It was suggested that the presence of the methyl group at the *para*-position of the phenyl ring allows the polymer chains to form an ordered structure. In fact, P(3HA-15 mol % 3H5pMPhV) also showed a *T*_m_ of 60 °C and *T*_g_ of 14 °C. However, the Δ*H*_m_ for this polymer is less than 1 J·g^−1^ [32], indicating that this sample is partially crystalline but highly amorphous. On the other hand, P(3H5PhV-64 mol % 3H5pMPhV), a PHA composed of monomers bearing phenyl- and methylphenyl-groups, is none crystalline [21], indicating that the 3H5PhV unit inhibits formation of the 3H5pMPhV crystalline structure.

#### 3.6.4. Thermal Properties of PHAs Containing Methylphenoxy Groups

P(3H4pMPhB-71.5 mol % 3H6pMPhHx), a PHA consisting of monomers containing the *para*-methylphenoxy group, is crystalline [33]. Precipitated samples of this polymer exhibit the typical spherulitic structure under a polarizing microscope. On the other hand, the Δ*H*_m_ of P(3H4mMPhB-70 mol % 3H6mMPhHx) is 0.2 J·g^−1^, indicating low crystallinity. These results indicate that the position of methyl substitution has a profound effect on the crystallinity of the polymers. P(3H5PhV-6.7 mol % 3H4pMPxB-28.3 mol % 3H6pMPxHx), a PHA consisting of monomers containing methylphenoxy- and phenyl-groups, is amorphous [33]. This indicates that the 3H5PhV unit inhibited the formation of a crystalline structure. This behavior is similar to that of aromatic PHAs bearing methylphenyl groups, as mentioned above.

#### 3.6.5. Thermal Properties of PHAs Containing Nitrophenyl Groups

From DSC analysis, P(3HA-6.9 mol % 3H5opNPhV), a PHA produced from 5-(*ortho*,*para*-dinitrophenyl)valeric acid and NA, showed one *T*_m_ and two *T*_g_ peaks [34]. The existence of two *T*_g_s indicates that the produced PHA is a mixture of two polymers [34]. The *T*_m_ of 56.42 °C and the *T*_g_ of −35.95 °C were assigned to the PHA produced from NA only, and the *T*_g_ of 28.74 °C is attributed to the newly synthesized polymer containing nitrophenyl rings. The lack of a defined *T*_m_ for this newly formed polymer indicates that the PHA containing the nitrophenyl group lacks crystalline domains and is thus amorphous.

#### 3.6.6. Thermal Properties of PHAs Containing Cyanophenoxy Groups

Incorporation of the 3H6pCPxHx unit into P(3HA) led to a slight decrease in the Δ*H*_m_ and *T*_m_ [35]. This indicates that the 3H6pCPxHx unit was coexistent with other monomer units in the same polymer chain, and disrupted the crystalline organization of the *n*-alkyl side groups, resulting in depressed *T*_m_ values.

P(3HA-19.6 mol% 3H6pCPxHx) showed a new *T*_g_ at −21 °C that was not observed for the samples with 0–6.8 mol % of the 3H6pCPxHx unit. This indicates that the product with 19.6 mol % 3H6pCPxHx is not a random copolymer, but is heterogeneous, having chains and/or chain segments enriched with the 3H6pCPxHx repeating units. Additionally, these segments would have unique crystal structures as the original melting peak of the sample occurred at >64 °C, where no melting peak was observed for P(3HA).

#### 3.6.7. Thermal Properties of PHAs Containing Fluorophenoxy Groups

Generally, fluoropolymers exhibit good thermal resistance. PHAs with a fluorinated side group also exhibit the expected behavior, with *T*_m_s of 52 °C (monofluorinated P(3H5pFPxV-8.7 mol % 3H7pFPxHp)) and 102 °C (difluorinated P(3H5opFPxV)) [36]. The wide angle X-ray scattering (WAXS) diffraction patterns indicated that fluorophenoxy PHA was crystalline, whereas phenoxy PHA without the fluorine atom was highly amorphous [36]. Incorporation of fluorine atoms into the side group clearly has a significant effect.

#### 3.6.8. Thermal Properties of PHAs Containing Thiophenoxy Groups

P(3H5TPxV-3H7TPxHp), a PHA consisting of thiophenoxy monomer units, has a *T*_g_ of 4 °C with no clear melting peaks [37], indicating that the polymer is amorphous, as supported by the X-ray diffraction pattern.

#### 3.6.9. Thermal Properties of PHAs Containing Benzoyl Groups

The PHA containing 79.8% 3H5BzV and 3HA (as determined from the GC-MS peak area) is relatively hard at room temperature, which indicates that this polymer has characteristic thermal properties [38]. From DSC analysis, it was determined that the *T*_g_ was 36 °C and the *T*_m_ was 150 °C. These values are higher than those of other aromatic PHAs. Generally, a high molecular weight can lead to high *T*_g_ and/or *T*_m_ values, but mcl-PHA (number-average molecular weight *M*_n_ = 170,000, weight-average molecular weight *M*_w_ = 350,000) and PHB (*M*_n_ = 1,800,000, *M*_w_ = 2,600,000), which have relatively high molecular weights, do not exhibit such high *T*_g_ or *T*_m_ values. Therefore, it was suggested that the incorporation of 79.8% of the 3H5BzV unit resulted in these high values. However, the thermal properties of other PHAs bearing benzoyl groups were similar to those of the other aromatic PHAs [38]. Thus, it could be concluded that the length of the side chain containing the benzoyl group and the number of monomeric units also influenced the thermal properties.

## 4. Effect of Incorporation of Aromatic Monomer on *T*_g_ of PHAs

As described in Section 3, the incorporation of aromatic monomers into PHA chains results in a distinct change in the thermal behavior. With an increase in the number of phenyl side groups introduced into P(3HB), the *T*_m_ and Δ*H*_m_ decreased, whereas the *T*_g_ increased. This trend in the *T*_g_ is much different from the cases of aliphatic monomers. The relationship between the aromatic monomer content and the *T*_g_ has been studied detail [15,17].

The *T*_g_ of a copolymer (*T*_gr_) can be calculated by applying the Fox equation, using the *T*_g_ of the constituent polymers (*T*_g1_ and *T*_g2_) [61]:

1/*T*_gr_ = W_1_/*T*_g1_ + W_2_/*T*_g2_ (1)

where the *T*_g_ values of the homopolymers are given in Kelvin, and W_1_ and W_2_ are the weight fractions of the respective polymers in the copolymer.

In the literature [15], from three experimental data sets (P(3HB) (*T*_g_ = 280.9 K, W_3H3PhP_ = 0), P(3HB-4.1 mol% 3H3PhP) (*T*_g_ = 283.7 K, W_3H3PhP_ = 0.068), and P(3HB-8.9 mol% 3H3PhP) (*T*_g_ = 287.6 K, W_3H3PhP_ = 0.143)), the *T*_g_ of the P(3H3PhP) homopolymer was estimated to be 62 °C (335 K). This estimated value is much higher than ambient temperature and is very close to that of poly(lactide) (*T*_g_ = 60 °C) [62]. Additionally, it was revealed that the estimated *T*_g_ for P(3HB-3H3PhP) with 33 mol% 3H3PhP (W_3H3PhP_ = 0.45) is 30 °C, which is the same as the culture temperature for PHA production. PHAs with *T*_g_s exceeding ambient temperature will be produced as rigid amorphous polymers. If PHA synthases do not prefer to polymerize rigid amorphous PHA, the production of P(3HB-3H3PhP) with 3H3PhP fractions of over 33 mol% may be a challenging task [15].

**Figure 2:**
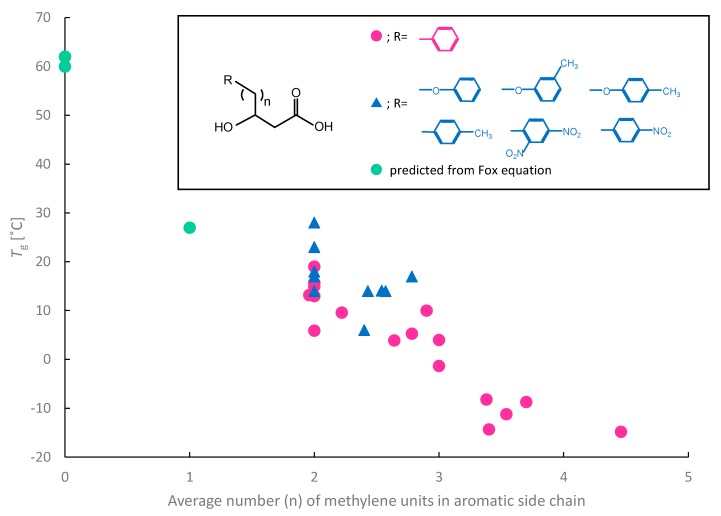
Correlation between *T*_g_ and average number of methylene units in aromatic side chain. Red circles: *T*_g_ of aromatic PHAs containing phenyl group; blue triangles: *T*_g_ of aromatic PHAs containing phenoxy, methylphenyl, methylphenoxy, or nitrophenyl group; green circles: *T*_g_ predicted from Fox equation. Values are from References [9,14,15,19,26,30,33,38,40,42,44,52,64].

The correlation between the average number of methylene units in the aromatic side chain and the *T*_g_ are shown in Figure 2, based on the literature data [15]. This figure shows the experimentally determined *T*_g_s and those predicted from the Fox equation. With increasing alkyl chain length of the aromatic side group on the monomers bearing the phenyl group, the *T*_g_ of the PHAs tends to decrease. This may be the result of an increase in the free volume. The rigidity of the side group further hampers the motion of the polymer backbone. Similar trends have been reported for mcl-PHAs [13], poly(alkyl methacrylate)s [63], and poly(alkyl itaconate)s [63]. In fact, the predicted *T*_g_ values from another study using the Fox equation were 60 °C for P(3H3PhP) and 27 °C for P(3H4PhB) [17], which are in good agreement with the foregoing data. Compared with 3H4PhB, the phenyl side group of 3H3PhP is smaller, but the rigidness of the side group is higher. Thus, 3H3PhP is more effective for increasing the *T*_g_ than 3H4PhB. These results suggest that the *T*_g_ of aromatic PHAs bearing phenyl groups depends not only on the size of the side group, but also on its rigidness.

As shown in Figure 2, most of the *T*_g_ values of aromatic PHAs bearing phenoxy, methylphenyl, methylphenoxy, and nitrophenyl groups followed trends similar to those of PHAs bearing the phenyl group.

## 5. Conclusions and Future Perspectives

The types of biosynthesized aromatic PHAs and their chemical and physical properties are summarized in this review. Various monomers containing phenyl, phenoxy, methylphenyl, methylphenoxy, nitrophenyl, nitrophenoxy, cyanophenoxy, fluorophenoxy, thiophenoxy, and benzoyl groups have been introduced into biosynthesized PHA chains. The chemical and physical properties of aromatic PHAs are different from those of aliphatic PHAs, and vary depending on the types of aromatic monomers. The *T*_g_ increases as the aromatic monomer content increases, which differs from the behavior of aliphatic monomers. Even with the introduction of a small amount of aromatic monomers, crystallization of P(3HB) is strongly inhibited. This behavior is attributed to the rigidity of the aromatic rings in the side groups. Additionally, the degradation rate of aromatic PHAs is slower than that of mcl-PHAs. This suggests that aromatic PHAs could be used as a drug vehicle which achieve slow release of the encapsulated drug.

The introduction of aromatic monomers broadens the range of applications by altering the properties of PHAs. Predicting the composition of PHAs that would provide the desired *T*_g_ using the Fox equation is a useful tool for controlling the properties of PHAs. The composition of PHAs can be controlled by changing the ratio of the carbon sources and by modification of the metabolic pathway, including the *β*-oxidation cycle of the microorganisms involved in PHA production [29,39].

For practical use of aromatic PHAs, certain issues must be addressed, such as the heterogeneity of the produced polymers, increasing the molecular weight of the PHAs, and reduction of the production cost. Under some conditions, aromatic PHAs are produced as heterogeneous polymers. Because the polymer homogeneity affects the material properties, the cultivation conditions should be optimized to produce homogeneous polymers rather than blend polymers. Additionally, increasing the molecular weight of PHAs is effective for improving the properties [65]. If the interaction between the aromatic rings can be strengthened by increasing the molecular weight, the physical and mechanical properties can be improved. As the types of PHA synthases affect the molecular weight of PHAs [66], the search for new PHA synthases (other than PHA synthases from *Pseudomonas*) that can polymerize aromatic monomers is a worthwhile undertaking. Reduction of the cost of the carbon sources and improving the productivity are critical approaches to reducing the production cost. The production of aromatic PHAs from biomass such as sugars without any precursor supplementation, which is generally expensive, would contribute to this end. However, the production of aromatic PHAs from sugars was just reported [16,18], so it may require additional research to achieve it. Utilization of biowastes as carbon source and co-production strategies would be beneficial methods for cost reduction [67].

All aromatic PHAs described above have aromatic rings in their side chains. The production of PHAs having aromatic rings in their backbone has not been reported to date, and achieving this structure would be a challenging task because there is no PHA synthase capable of polymerizing aromatic rings in the PHA backbone thus far. If PHAs having aromatic rings in the backbone can be produced, they may show good thermostability, similar to polyethylene terephthalate.

The incorporation of aromatic monomers is a promising method for improving the properties of PHAs and conferring physical properties superior to those of aliphatic PHAs. Further, this approach is not expected to compromise the important characteristics of PHAs, including their biocompatibility, biodegradability, and thermoplasticity. Thus, incorporation of aromatic monomers into the PHA chain is proposed as a promising method of improving the material properties of PHAs.

## Figures and Tables

**Table 1:** Cultivation conditions for production of aromatic polyhydroxyalkanoates (PHAs).

Aromatic Group		Polymer	Bacterial Strain	Carbon Substrate	Reference
Phenyl	(homopolymer)	P(3H5PhV)	*P. oleovorans*	5PhV	[14]
		P(3H5PhV)	*P. putida* BM01	5PhV	[19]
		P(3H5PhV)	*P. putida* KT2440	5PhV	[15]
		P(3H5PhV)	*P. oleovorans*	5-phenyl-2,4-pentadienoic acid	[20]
		P(3H6PhHx)	*P. putida* U	6PhHx	[25]
	(copolymer)	P(3H5PhV-3H7PhHp)	*P. putida* U	7PhHp	[26]
		P(3H6PhHx-3H8PhO)	*P. putida* U	8PhO	[25]
		P(3H6PhHx-3H8PhO-3H10PhD)	*P. putida* U	10PhD	[25]
	(copolymer containing aliphatic monomers)	P(3HA-3H5PhV)	*P. oleovorans*	5PhV, HA	[27]
		P(3HA-3H5PhV)	*P. citronellolis*	5PhV, OA	[28]
		P(3HA-HE-3H5PhV)	*P. putida* GPo1	5PhV, OA, 10-undecenoic acid	[29]
		P(3HA-3H4PhB-3H6PhHx)	*P. fluorescens* B3	6PhHx	[22]
		P(3HA-3H4PhB-3H7PhHx)	*P. jessenii* C8	7PhHx	[22]
		P(3HB-3HA-3H3PhP)	*R. eutropha* PHB^-^4 expressing PhaC1_Ps_	3-hydroxy-3-phenylpropionic acid, fructose	[15]
		P(3HB-3HA-3H3PhP)	*R. eutropha* PHB^-^4 expressing PhaC1_Ps_	cinnamic acid, fructose	[15]
		P(3HB-3HA-3H4PhB)	*R. eutropha* PHB^-^4 expressing PhaC1_Ps_	4PhB, fructose	[17]
		P(3HB-3HA-2H3PhP)	*E. coli* expressing mutated PhaC1_Ps_	phenylalanine, sugars	[16]
		P(3HB-3HA-2H3PhP)	*E. coli* expressing mutated PhaC1_Ps_	sugars	[16]
Phenoxy		P(3H5PxV-3H7PxHp)	*P. putida* BM01	11-phenoxyundecanoic acid	[30]
		P(3H4PxB-3H6PxHx-3H8PxO)	*P. oleovorans*	8-phenoxyoctanoic acid	[31]
		P(3H5PxV-3H7PxHp-3H9PxN)	*P. oleovorans*	11-phenoxyundecanoic acid	[31]
Methylphenyl		P(3H5pMPhV)	*P. oleovorans*	5-(*para*-methylphenyl)valeric acid	[21]
		P(3HA-3H5pMPhV)	*P. putida*	9-(*para*-methylphenyl)nonanoic acid	[32]
Methylphenoxy		P(3H4pMPxB-3H6pMPxHx) ^1^	*P. putida*	8-(*para*-methylphenoxy)octanoic acid	[33]
		P(3H4mMPxB-3H6mMPxHx)	*P. putida*	8-(*meta*-methylphenoxy)octanoic acid	[33]
Nitrophenyl		P(3HA-3H5(pNPh and/or opNPh)V)	*P. oleovorans*	5-(*ortho,para*-dinitrophenyl)valeric acid	[34]
Nitrophenoxy		PHA ^2^	*P. oleovorans*	OA, 6-(*para*-nitrophenoxy)hexanoic acid	[24]
Cyanophenoxy		P(3HA-3H6pCPxHx)	*P. putida* KT2440	OA, 6-(*para*-cyanophenoxy)hexanoic acid	[35]
		P(3HHx-3HO-3H5pCPxV)	*P. putida* KT2442	OA, 5-(*para*-cyanophenoxy)valeric acid	[24]
Fluorophenoxy		P(3H5mFPxV-3H7mFPxHp)	*P. putida*	11-(*meta*-fluorophenoxy)undecanoic acid	[36]
		P(3H5pFPxV-3H7pFPxHp)	*P. putida*	11-(*para*-fluorophenoxy)undecanoic acid	[36]
		P(3H5opFPxV)	*P. putida*	11-(*ortho,para*-difluorophenoxy)undecanoic acid	[36]
Thiophenoxy		P(3H5TPxV-3H7TPxHp)	*P. putida* 27N01	11-thiophenoxy undecanoic acid	[37]
Benzoyl		P(3HA-3H4BzB)	*P. cichorii* YN2	4-benzoylbutyric acid	[38]
		P(3H5BzV-3H7BzHp)	*P. cichorii* YN3	7-benzoylheptanoic acid	[38]
		P(3H5BzV-3H6BzHx-3H8BzO)	*P. cichorii* YN4	8-benzoyloctanoic acid	[38]

^1^ A small amount of the 3H8pMPxO unit was also detected by GC analysis. ^2^ PHA containing nitrophenoxy group.

**Table 2:** Mechanical properties of P(3HDD-3H5PhV) with various 3H5PhV contents.

Polymer ^1^	Mechanical Properties												Molecular Weight ^2^			Reference
	Yield Strength (MPa)			Maximum Tension Strength (MPa)			Elongation at Break (%)			Young’s Modulus (MPa)			*M*_n_ (10^4^)	*M*_w_ (10^4^)	*M*_w_/*M*_n_	
P(3HDD)	5.5	±	0.8	5.5	±	0.9	60	±	34	61.1	±	6.4	5.2	10.4	2.0	[52]
P(3HDD-2.91 mol% 3H5PhV)	1.53	±	0.65	2.05	±	0.51	37.38	±	6.28	93.91	±	20.52	4.1	6.56	1.6	[52]
P(3HDD-18.7 mol% 3H5PhV)	3.63	±	0.68	4.36	±	0.94	86.03	±	39.80	94.79	±	34.95	4.3	7.31	1.7	[52]
P(3HDD-31.9 mol% 3H5PhV)	2.84	±	1.05	3.15	±	1.21	32.02	±	15.94	48.72	±	24.04	3.4	6.12	1.8	[52]
P(3H5PhV)	― ^3^			― ^3^			― ^3^			― ^3^			2.1	4.41	2.1	[52]
P(3HB) produced by wild-type bacteria		-		43			5			3500			-	1–300	approx. 2	[53]
Polypropylene		-		38			400			1700			-	-	-	[53]
Low-density polyethylene		-		10			620			200			-	-	-	[53]

^1^ 3HDD, 3-hydroxydodecanoate; 3H5PhV, 3-hydroxy-5-phenylvalerate. ^2^ *M*_n_, number-average molecular weight; *M*_w_, weight-average molecular weight. ^3^ The sample was sticky even at room temperature. “-” indicate “data was not presented”.

**Table 3:** Thermal properties of aromatic PHAs.

Aromatic Group	Polymer	Thermal Properties ^4^			Molecular Weight ^5^		Reference
		*T*_m_ (°C)	*T*_g_ (°C)	Δ*H*_m_ (J/g)	*M*_n_ (10^4^)	*M*_w_/*M*_n_	
―	P(3HB)	162, 178	5	51	32	1.9	[17]
	mcl-PHA	45 to 69	−53 to −28	-	17	2.1	[13]
	Polypropylene	176	−10	-	-	-	[53]
	Low-density polyethylene	130	−30	-	-	-	[53]
Phenyl	P(3H5PhV)	54 to 69	13	-	10	3.5	[14]
(homopolymer)	P(3H6PhHx)	not detectable	−1.3	-	21.6	2.2	[26]
	P(3H4PhB-95 mol% 3H6PhHx)	not detectable	10	-	-	-	[19]
Phenyl	P(3H6PhHx-73 mol% 3H8PhO)	not detectable	−14.8	-	8.2	2.0	[26]
(copolymer)	P(3H5PhV-77 mol% 3H7PhHp)	not detectable	−11.2	-	6.7	2.3	[26]
	P(3H5PhV-38 mol% 3H6PhHx-50 mol% 3H7PhHp)	not detectable	−8.2	-	13.8	2.7	[26]
Phenyl (copolymer containing aliphatic monomers)	P(95.1 mol% 3HB-4.1 mol% 3H3PhP)	146, 158	10.7	27.8	6.5	3.5	[15]
	P(89.5 mol% 3HB-8.9 mol% 3H3PhP)	135, 149	14.6	7.1	9.7	3.7	[15]
	P(3HA-98 mol% 3H5PhV)	51.5	13.2	-	2.5	3.1	[42]
	P(3HA-15 mol% 3H4PhB-83 mol% 3H6PhHx)	52.1	3.9	-	9.1	3.5	[42]
	P(3HA-85 mol% 3H5PhV-13 mol% 3H7PhHp)	not detectable	9.6	-	6.7	3.1	[42]
	P(3HA-7 mol% 3H4PhB-61 mol% 3H6PhHx-30 mol% 3H8PhO)	not detectable	−14.3	-	7.2	4.1	[42]
	P(3HA-6 mol% 3H4PhB-57 mol% 3H6PhHx-26 mol% 3H8PhO-9 mol% 3H10PhD)	not detectable	−8.7	-	5.7	2.9	[42]
	P(3HA-45 mol% 3H5PhV)	-	−20	-	-	-	[28]
	P(3HA-40.6 mol% 3H5PhV) ^1^	49	5, −31	7.2	5	2.0	[27]
	P(3HA-10 mol% HE-59 mol% 3H5PhV)	not detectable	−6	-	12.4	2.9	[29]
	(P(3HA-10 mol% 3HE)	40.1	−38.7	-	8.6	2.2	[29])
	P(3H5PhV)	50.4	5.9	-	2.1	2.1	[52]
	P(3HDD-31.97 mol% 3H5PhV)	75.84	−35.15	-	3.4	1.8	[52]
	P(3HDD-18.7 mol% 3H5PhV)	80.13	−35.81	-	4.3	1.7	[52]
	P(3HDD-2.91 mol% 3H5PhV)	81	−33.35	-	4.1	1.6	[52]
	(P(3HDD)	82.4	−49.3	-	5.2	2.0	[52])
	P(3HB-12 mol% 3H3PhP)	132, 148	9	4	18	3.5	[17]
	P(3HB-15 mol% 3H3PhP)	not detectable	15	not detectable	14	2.4	[17]
	P(3HB-18 mol% 3H3PhP)	not detectable	16	not detectable	9.2	2.1	[17]
	P(3HB-21 mol% 3H3PhP)	not detectable	20	not detectable	3.8	2.1	[17]
	P(3HB-4 mol% 3H4PhB)	138, 151	7	46	7.1	2.0	[17]
	P(3HB-8 mol% 3H4PhB)	126, 136	9	12	4.5	2.4	[17]
	P(3HB-15 mol% 3H4PhB)	105, 119	10	3	1.7	2.3	[17]
Phenoxy	P(3H5PxV-27 mol% 3H7PxHp)	70	14.1	2.9	-	-	[30]
	P(3H5PxV)	88	23	-	7.4	2.0	[38]
Methylphenyl	P(3H5pMPhV)	95	18	-	-	-	[21]
	P(3HA-15 mol% 3H5pMPhV)	60	14	0.1	5	2.4	[32]
Methylphenoxy	P(3H4pMPxB-71.5 mol% 3H6pMPxHx)	97	14	33.5	2.5	2.5	[33]
	P(3H5PhV-6.7 mol% 3H4pMPxB-28.3 mol% 3H6pMPxHx)	not detectable	17	-	-	-	[33]
	P(3H4mMPxB-70 mol% 3H6mMPxHx)	43	6	0.2	-	-	[33]
Nitrophenyl	P(3HA-6.9 mol% 3H5(pNPh and/or opNPh)V)	not detectable	28.74	-	-	-	[34]
Cyanophenoxy	P(3HA-19.6 mol% 3H6pCPxHx) ^1^	53.5, >64	−37.5, −21	15.1	-	-	[35]
	(P(3HA)	55.5	−35.4	19.3	-	-	[35]
Fluorophenoxy	P(3H5opFPxV)	102	-	-	1.3	2.8	[36]
	P(3H5pFPxV-8.7 mol% 3H7pFPxHp)	52	-	-	1.1	1.9	[36]
Thiophenoxy	P(3H5TPxV-3H7TPxHp) ^2^	not detectable	4	-	8.1	1.8	[37]
Benzoyl	P(3HA-79.8%3H5BzV) ^3^	150	36	-	33	3.9	[38]

^1^ The sample comprised a mixture of two PHAs (see text). ^2^ 3H5TPxV is the primary monomer unit. ^3^ The monomer ratio was determined from the peak area from GC-MS analysis. ^4^ *T*_m_, melting temperature; *T*_g_, glass transition temperature; Δ*H*_m_, enthalpy of fusion. ^5^ *M*_n_, number-average molecular weight; *M*_w_, weight-average molecular weight. “-” indicate “data was not presented”.

## Abbreviations

The following abbreviations are used in this manuscript.

PHAs	Polyhydroxyalkanoates
P(3HB)	poly(3-hydroxybutyrate)
mcl-PHA	medium-chain-length PHA
3HA	3-hydroxyalkanoate
3HE	3-hydroxy-*ω*-alkenoate
3HPhA	3-hydroxy-*ω*-phenylalkanoate
3HHx	3-hydroxyhexanoate
3HHp	3-hydroxyheptanoate
3HO	3-hydroxyoctanoate
3HN	3-hydroxynonanoate
3HD	3-hydroxydecanoate
3HDD	3-hydroxydodecanoate
OA	octanoic acid
NA	nonanoic acid
4PhB	4-phenylbutyric acid
5PhV	5-phenylvaleric acid
6PhHx	6-phenylhexanoic acid
7PhHp	7-phenylheptanoic acid
8PhO	8-phenyloctanoic acid
10PhD	10-phenyldecanoic acid
2H3PhP	2-hydroxy-3-phenylpropionate
2H4PhB	2-hydroxy-4-phenylbutyrate
3H3PhP	3-hydroxy-3-phenylpropionate
3H4PhB	3-hydroxy-4-phenylbutyrate
3H5PhV	3-hydroxy-5-phenylvalerate
3H6PhHx	3-hydroxy-6-phenylhexanoate
3H7PhHp	3-hydroxy-7-phenylheptanoate
3H8PhO	3-hydroxy-8-phenyloctanoate
3H10PhD	3-hydroxy-10-phenyldecanoate
3H4PxB	3-hydroxy-4-phenoxybutyrate
3H5PxV	3-hydroxy-5-phenoxyvalerate
3H6PxHp	3-hydroxy-6-phenoxyhexanoate
3H7PxHp	3-hydroxy-7-phenoxyheptanoate
3H8PxO	3-hydroxy-8-phenoxyoctanoate
3H9PxN	3-hydroxy-9-phenoxynonanoate
3H5pMPhV	3-hydroxy-5-(*para*-methylphenyl)valerate
3H4pMPxB	3-hydroxy-4-(*para*-methylphenoxy)butyrate
3H6pMPxHx	3-hydroxy-6-(*para*-methylphenoxy)hexanoate
3H8pMPxO	3-hydroxy-8-(*para*-methylphenoxy)octanoate
3H4mMPxB	3-hydroxy-4-(*meta*-methylphenoxy)butyrate
3H6mMPxHx	3-hydroxy-6-(*meta*-methylphenoxy)hexanoate
3H5pNPhV	3-hydroxy-5-(*para*-nitrophenyl)valerate
3H5opNPhV	3-hydroxy-5-(*ortho*,*para*-nitrophenyl)valerate
3H5pCPxV	3-hydroxy-5-(*para*-cyanophenoxy)valerate
3H6pCPxHx	3-hydroxy-6-(*para*-cyanophenoxy)hexanoate
3H5mFPxV	3-hydroxy-5-(*meta*-fluorophenoxy)valerate
3H7mFPxHp	3-hydroxy-7-(*meta*-fluorophenoxy)heptanoate
3H5pFPxV	3-hydroxy-5-(*para*-fluorophenoxy)valerate
3H7pFPxHp	3-hydroxy-7-(*para*-fluorophenoxy)heptanoate
3H5opFPxV	3-hydroxy-5-(*ortho*,*para*-fluorophenoxy)valerate
3H5TPxV	3-hydroxy-5-thiophenoxyvalerate
3H7TPxHp	3-hydroxy-7-thiophenoxyheptanoate
3H4BzB	3-hydroxy-4-benzoylbutyrate
3H5BzV	3-hydroxy-5-benzoylvalerate
3H6BzHx	3-hydroxy-6-benzoylhexanoate
3H7BzHp	3-hydroxy-7-benzoylheptanoate
3H8BzO	3-hydroxy-8-benzoyloctanoate
*T*_g_	glass transition temperature
*T*_m_	melting temperature
Δ*H*_m_	enthalpy of fusion
*M*_n_	number-average molecular weight
*M*_w_	weight-average molecular weight
